# Early Detection of Radiation-Induced Injury and Prediction of Cognitive Deficit by MRS Metabolites in Radiotherapy of Low-Grade Glioma

**DOI:** 10.1155/2021/6616992

**Published:** 2021-03-04

**Authors:** Zahra Alirezaei, Alireza Amouheidari, Masoud Hassanpour, Fariba Davanian, Sajjad Iraji, Parvaneh Shokrani, Mohammad-Reza Nazem-Zadeh

**Affiliations:** ^1^Medical Physics Department, Isfahan University of Medical Science, Isfahan, Iran; ^2^Isfahan Milad Hospital, Deputy Manager in Research & Education, Head, Department of Radiation Oncology, Isfahan, Iran; ^3^Research Center for Molecular and Cellular Imaging, Tehran University of Medical Sciences, Tehran, Iran; ^4^Radiology Department, School of Medicine, Isfahan University of Medical Science, Isfahan, Iran; ^5^Medical Physics and Biomedical Engineering Department, School of Medicine, Tehran University of Medical Sciences, Tehran, Iran; ^6^Research Center for Molecular and Cellular Imaging, Advanced Medical Technology and Equipment Institute, Tehran University of Medical Sciences, Tehran, Iran

## Abstract

**Purpose:**

To compare the sensitivity of MRS metabolites and MoCA and ACE-R cognitive tests in the detection of radiation-induced injury in low grade glioma (LGG) patients in early and early delayed postradiation stages.

**Methods:**

MRS metabolite ratios of NAA/Cr and Cho/Cr, ACE-R and MoCA cognitive tests, and dosimetric parameters in corpus callosum were analyzed during RT and up to 6-month post-RT for ten LGG patients.

**Results:**

Compared to pre RT baseline, a significant decline in both NAA/Cr and Cho/Cr in the corpus callosum was seen at the 4th week of RT, 1, 3, and 6-month post-RT. These declines were detected at least 3 months before the detection of declines in cognitive functions by ACE-R and MoCA tools. Moreover, NAA/Cr alterations at 4th week of RT and 1-month post-RT were significantly negatively correlated with the mean dose received by the corpus callosum, as well as the corpus callosum 40 Gy dose volume, i.e., the volume of the corpus callosum receiving a dose greater than 40 Gy.

**Conclusion:**

MRS-based biomarkers may be more sensitive than the state-of-the-art cognitive tests in the prediction of postradiation cognitive impairments. They would be utilized in treatment planning and dose sparing protocols, with a specific focus on the corpus callosum in the radiation therapy of LGG patients.

## 1. Introduction

Radiation therapy (RT) plays a major role in treatment of low-grade glioma (LGG) patients. During RT, the brain normal tissue will inevitably be exposed to the radiation, causing detrimental effects called radiation-induced brain injury (RIBI) [1, 2]. Depending on the time between the onset of RT and the clinical manifestations, the effects are classified as early, early delayed, and late delayed.

Early effects occur during or shortly after the RT and characterized by symptoms of fatigue, dizziness, and increased intracranial pressure [3, 4]. Early delayed injuries occurring one to six months after RT generally show reversible symptoms such as global weakness and somnolence, resulting from a temporary demyelination. The more severe late delayed brain injury usually develops in and about 6-month postradiation. These injuries may lead to severe irreversible neurological consequences without any visible sign on conventional neuroimaging and histopathology [2, 3, 5]. In long-term surviving patients, the late delayed effects lead to cognitive impairments including disabilities in learning, working memory, executive function, vision, motor functions, and dementia [2].

Pathophysiology of radiation-induced injury of central nervous system (CNS) is poorly understood [5], but there are reports that it may affect anatomic and functional structures in the cerebellum, frontal, and temporal lobes, by creating vascular abnormalities, inflammation, gliosis, demyelination, and white matter necrosis [6–10]. Late delayed radiation induced damages have been reported as alterations in the morphology and integrity of white matter detected in conventional MRI [11]. However, radiation-induced microstructural changes that may later lead to cognitive impairments are evidenced to emerge in early and early delayed phases. These changes may occur without radiographic or clinical manifestation of demyelination or white matter necrosis in later phases [12] and can be detectable by diffusion tensor imaging (DTI) [13–15] in early and early delayed phases [16].

MR spectroscopy (MRS) can evaluate RIBI by assessing the metabolic concentrations at the molecular levels [9, 17]. Variation of three important metabolites including N-acetylaspartate (NAA), choline (Cho), and creatine (Cr) in MRS [18–21] has been inspected in many studies to evaluate RIBI. The NAA metabolite predominantly present in the neurons is recognized as a marker for neural cell density and neuronal function [18]. NAA variation might be especially important for the speed of cognitive processing [3]. Choline compound, on the other hand, is associated with membrane biosynthesis and metabolic activity in proliferative tissue [22]. Creatine, in contrast, is renowned as a marker for cellular energy metabolism, consented to be fairly stable under different pathophysiological conditions [17–20].

The corpus callosum (CC) is the largest white matter commissural bundle that interconnects the right and left brain hemispheres at the genu, the main body, and the splenium [23–25]. The CC connects the sensory-motor and association cortices of the cerebral hemispheres associated with intellectual performance [4]. Verbal fluency and language lateralization correlated with the splenium of CC. Also, visuospatial construction abilities, language, and psychomotor function are associated with the CC [10]. Depending on its specific anatomical location, lesions or morphological alterations in the CC can lead to the Wallerian degeneration [26, 27], as well as cognitive impairments including slow processing, complications with visuospatial perception, dysnomia, and other cognitive impairments [23–25, 28]. The CC is evidenced to be sensitive to the radiation effects [13–15] leading to alterations in cognitive functions [17], yet a dose-sparing protocol has not sufficiently considered during conventional radiation treatment planning [9, 10].

The relationship between morphological and geometric alterations in the CC and cognitive impairments in infarction, traumatic brain injury (TBI), and neurodegenerative diseases such as Alzheimer disease (AD), Parkinson's disease (PD), schizophrenia (SZ), and dementia has been established using cognitive assessments tools such as the Montreal Cognitive Assessment (MoCA) tools, Addenbrooke Cognitive Examination (ACE), and Mini-Mental Status Examination (MMSE) as an abbreviated version of ACE [29–31]. The MoCA has been successfully employed to assess the cognitive functioning of patients with brain metastases after radiotherapy [32]. ACE Revised Version (ACE-R) is an established battery of tasks used for clinical practice [30, 31]. It covers various cognitive domains, namely, memory, language and verbal fluency, attention and orientation, and visuospatial perception [33]. ACE-R is comparable to MoCA, and there is a significant correlation of scores between the two batteries [34]. Some works have found ACE-R superior than MoCA in terms of sensitivity and specificities in the diagnosis of mild cognitive impairments, and it is regarded as a gold standard for the screening and diagnostic accuracy of cognitive impairment [35].

The changes in the concentration of MRS metabolites in the CC have been also associated with psychopathology in these diseases [36]. MRS investigations of proton metabolites in CC suggest that individual variation in cognitive function can be predicted by NAA/Cr and Cho/Cr ratios, namely, a reduction of Cho/Cr and NAA/Cr in AD and SZ patients [37]. In addition, a decline in NAA/Cr and Cho/Cr ratios during and after RT of the doses as low as 20 Gy [31] is often indicative of cell damage or cell death due to apoptosis as a secondary defect in the function of nerve cells [24, 25].

Post-RT cognitive impairments are not commonly detectable by cognitive assessment tools such as the MMSE, ACE, and MoCA [38–40] in early delayed phase. Moreover, the accuracy of these assessment tools in estimating the cognitive states cannot be confidently validated due to many sources of variability such as assessment time and the grade and location of the tumors [38].

This study is aimed at investigating whether the change in MRS metabolite can be a more sensitive tool for detecting brain injury in early post-radiation phase compared to conventional cognitive tools.

To accomplish this aim, MRS metabolites and MoCA and ACE-R subdomain scores were analyzed at different time points before, during, and after radiotherapy. The correlations between these parameters and the delivered radiation dose to corpus callosum were investigated.

## 2. Material and Methods

### 2.1. Study Design

This prospective study included 10 patients (5 males and 5 females; age range 19–55 years; mean age, 36.25 years) histologically confirmed with oligodendroglioma WHO GII with IDH mutant and 1p/19q codeletion as the genetic factor biomarker of this type of tumor (*n* = 10). All patients had seizure at base line and were prescribed with antiepileptic drugs (Table 1).

### 2.2. Treatment Planning and Dosimetry

All patients underwent craniotomy and RT without receiving chemotherapy. Standard 3D conformal (3D-CRT) fractionated partial brain RT was prescribed using 1.8 Gy in 30 fractions by ONCOR linear accelerators (Linac; Siemens Healthcare, Erlangen, Germany). Dosimetric parameters of the CC were extracted from the dose volume histograms (DVH) computed using the Prowess Panther planning system version 5.5 (Prowess Inc., Concord, CA). Maximum dose (*D*_max_), minimum dose (*D*_min_), and mean dose (*D*_mean_) received by the CC were 54.7 ± 17.5 Gy, 18.2 ± 7.6 Gy, and 34.1 ± 8.7 Gy, with the volume of 8.1 ± 1.5 CC received dose more than 40 Gy.

### 2.3. MRI and MRS Image Acquisition

**Figure 1:**
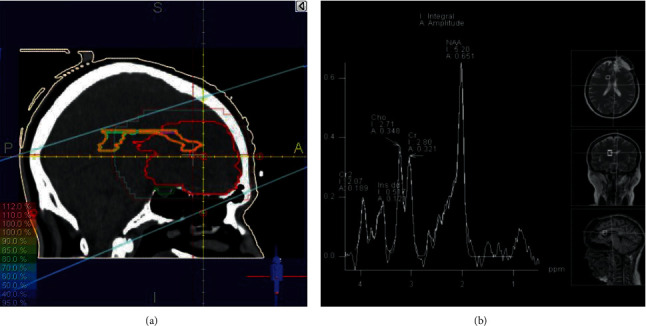
(a) Isodose curves of a treatment plan of a LGG patient with a left frontal tumor on Prowess Panther 5.5. (b) An example of MRS metabolite peaks of a voxel on the CC, fused with T2W MRI on a 1.5 Tesla Siemens Magnetom Aera scanner.

All patients had MRI/MRS scans (1.5 Tesla Magnetom Aera scanner, Siemens Healthcare Headquarters, Erlangen, Germany) at the baseline (before RT) and at the fourth week of RT, 1, 3, and 6-month after RT. The conventional MRI brain scan protocol included sagittal and axial pre- and postgadolinium T1-weighted images, axial T2-weighted FLAIR (fluid attenuated inversion recovery), T2-weighted images, and post-gadolinium T1-weighted coronal images. Spectroscopic data were acquired using multivoxel 2D-CSI (Chemical Shift Imaging) with the following parameters: TE/TR 135/2000 msec, field of view (FOV) 160∗160 mm^2^, thickness 15 mm, matrix 16 × 16, 1 NEX, with use of saturation bands outside the FOV. The PRESS (Point Resolved Spectroscopy) region centered on the CC was chosen large enough (8 × 8 cm^2^) to recover the whole CC (Figure 1). The metabolic spectra of the 2D CSI were analyzed using the jMRUI v.5.2 software by manual adjustment of metabolic peak boundaries [41]. For each patient, the spectra from at least 15 voxels of CC were selected. The metabolic ratios of NAA/Cr and Cho/Cr were calculated by analyzing the areas under the metabolite spectra.

### 2.4. Cognitive Tests

The Farsi versions of the (MoCA-P) [42] and (ACE-P) [43] were completed by all patients at the time of their MRI scans. MoCA-P contains questions measuring the following cognitive domains: visuospatial/executive function, naming, memory, language, abstraction, and attention.

ACE-R included five subfactors to assess different cognitive functions with the maximum score of 100; each allocated to one of the following sub-factors: attention/orientation [18], memory [26], fluency [14], language [26], and visuospatial ability [16]. A cutoff point of 84 for Persian ACE-R was considered to establish the normal status, to which a sensitivity of 93% and specificity of 91% has been assigned when discriminating MCI from a normal population [44].

### 2.5. Statistical Analysis of MR Spectroscopy and Cognitive Tests

The significance of sequential deviations from the baseline values for the metabolic ratios of Cho/Cr and NAA/Cr and the ACE-R (total and subdomains) and MoCA (total and subdomains) results were inspected using a paired-sample Student's *t* test. All tests were two-tailed and conducted at the 5% significance level. Considering the comparison for 4 MRS measures and cognitive tests in 4 follow-up time points with the baseline value, a multiple comparison was addressed by Bonferroni adjustment, by dividing the significance level of the statistical test by 16 (*α* = 0.05/16 = 0.003).

The correlations between the metabolic ratios and ACE-R and MoCA (total and subdomains) scores were assessed by Pearson's correlations. The correlation between the alterations in NAA/Cr and Cho/Cr in with the alterations in ACE-R (total and subdomains) and MoCA (total and subdomains) scores in consecutive time points was also inspected.

## 3. Results

All patients had stable disease, i.e., no interval change in tumor size, configuration, contrast enhancement, or peritumoral edema in follow-up periods following the fourth week of RT.

### 3.1. MRI Findings

Among all patients studied, no definite radiation-induced lesion was evident by visual inspection of T2-weighted, FLAIR, and postgadolinium T1-weighted images up to 6 months after RT. All patients had hyperintense signals on T2-weighted FLAIR images within the tumor and its periphery that did not significantly change during the 6-month follow-up. Also, there was no enhanced signal on the normal appearing of the CC for eight patients during 6-month follow-up. In one patient, mild scattered focal areas of increased T2/FLAIR signal abnormalities were present in the white matter of parietal lobe adjacent to the CC genu and periventricular white matter one month after RT, consistent with old ischemic changes. These areas demonstrated reduced enhancement over the 1-month follow-up and disappeared on the 3- and 6-month follow-ups. No new areas of signal abnormalities or pathological contrast enhancement suggestive of radiation-induced abnormalities were present in the follow-up images. In another patient with mixed glioma, two heterogeneously enhanced foci were seen in the white matter and specifically the CC at the 3-month follow-up.

### 3.2. MRS Findings

**Figure 2:**
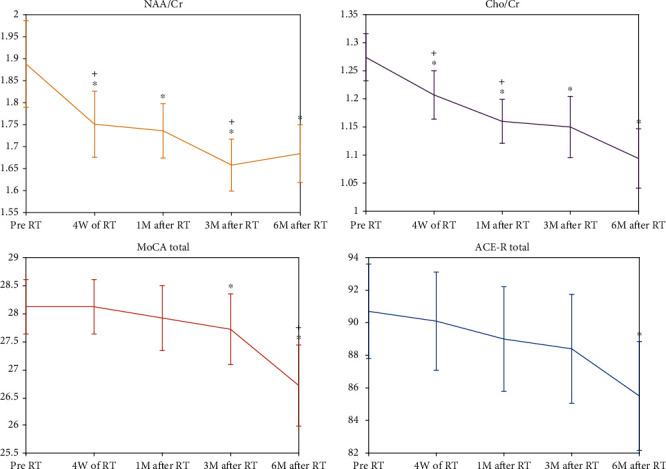
Plots of the standard error of the mean values of NAA/Cr and Cho/Cr, along with the ACE-R and MoCA scores at the baseline, 4^th^ week of RT, 1-month, 3-month, and 6-month post-RT values. The star markers (∗) show significant differences to the base line, while the plus ones (+) present the significant differences between any parameters to its previous time point.

Only one patient was missed at the 3-month follow-up scan. All other patients successfully fulfilled all follow-up scans. The values of NAA/Cr and Cho/Cr ratios in normal-appearing brain tissue of the CC prior to the RT were measured as 1.89 ± 0.29 and 1.27 ± 0.12, respectively (mean ± SD). Compared to the pre-RT values, the NAA/Cr ratio decreased by about 10%, 12%, 17%, and 18% at the fourth week of RT, 1-month, 3-month, and 6-month post-RT, respectively (*P* < 0.05, Figure 2). All decrements stayed significant after Bonferroni adjustment, except for the fourth week of RT. Again, compared to the pre-RT values, the mean of the Cho/Cr ratio decreased by about 12%, 15%, 24%, and 20% at the fourth week of RT, 1-month, 3-month, and 6-month post-RT, respectively (*P* < 0.05, Figure 2). Again, all decrements stayed significant after Bonferroni adjustment except for the fourth week of RT.

Inspecting the temporal variations in MRS metabolites (Figure 2) showed significant difference for NAA/Cr values at the fourth week of RT compared to the baseline (*P* = 0.001); and at the 3-month compared to 1-month follow-up (*P* = 0.001). The value of Cho/Cr, on the other hand (Figure 2), showed significant variation at the fourth week of RT compared to the baseline and at the fourth week of RT compared to the 1-month follow-up (*P* = 0.0001).

### 3.3. Cognitive Tests

Compared to the baseline values, both the MoCA and ACE-R scores taken 6-month post RT were statistically significant (*P* = 0.0001 and 0.002, respectively, Figure 2). No other time point scores were significantly different with baseline values. The MoCA scores at 6-month scores were significantly dropped compared to 3-month scores (*P* = 0.0001, Figure 2).

At baseline, the scores of MoCA and ACE-R tests were below the normal thresholds (MoCA < 26, ACE-R < 84) for one and three patients, respectively. For two patients, the scores of MoCA were measured below the normal (<26) at 3-month and 6-month post-RT. However, the ACE-R scores of three patients were below the normal (<84) at the baseline, as well as any other time points. Overall, five and four patients had ACE-R and MoCA scores below the normal at 6-month post-RT, respectively (four patients were common between the test).

**Figure 3:**
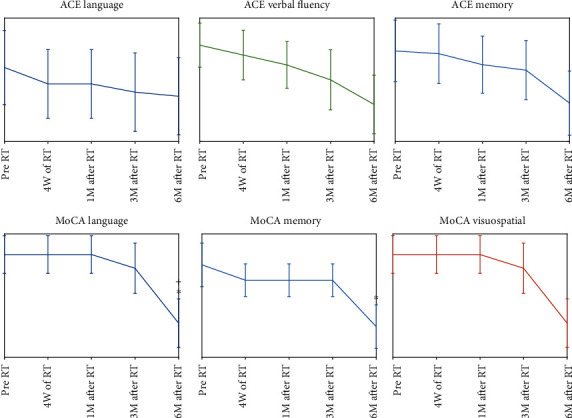
Plots of the standard error of the mean values of ACE-R (language, verbal fluency, and memory) and MoCA domain scores (language, memory, and visuospatial) at the baseline and 4^th^ week of RT, 1-month, 3-month, and 6-month post-RT. The star markers show significant differences to the baseline, while the plus ones present the significant differences between any parameters to its previous time point.

A significance decline was observed in MoCA language domain at 6-month compared to 3-month post-RT (*P* value = 0.05, before Bonferroni adjustment, Figure 3). Also, there was a significant difference between MoCA language score at the 6-month follow-up compared to the baseline value (*P* value = 0.045, before Bonferroni adjustment, Figure 3). Furthermore, a significant decrease was seen between the scores of MoCA memory subdomain at the 6-month post-RT to the baseline value (*P* value = 0.04, before Bonferroni adjustment, Figure 3). Although none of these declines stayed significant after Bonferroni adjustment, they still show a trend of declines. According to Table 2, ACE-R showed declines in language and verbal fluency subdomains for two patients at the 4^th^ week of RT. ACE-R detected declines after 1-month and 3-month post-RT, for two patients and one patient, respectively. However, both ACE-R and MoCA showed a decline by both the language and verbal fluency subdomains at 6-month post-RT. Overall, ACE-R and MoCA tests showed cognitive declines for 6 patients, 6-month post-RT.

**Figure 4:**
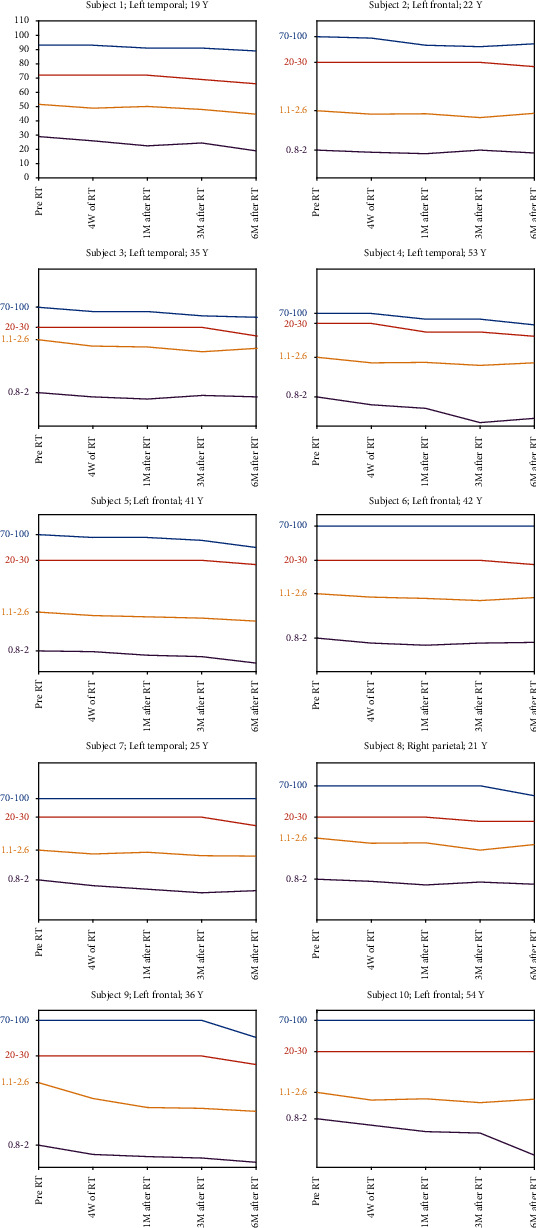
The interval variation of NAA/Cr, Cho/Cr, ACE-R, and MoCA (total scores), from pre-RT up to 6-month post-RT for individual patients. The patient's age along with the location of tumor is indicated above the graphs.

According to Table 2 and Figure 4, ACE-R and MoCA altogether showed temporal trends of decline in cognitive functions, ending up with 6, 5, and 5 patients with severe impairments in memory, language and verbal fluency, and visuospatial domains, respectively, at 6-month post-RT.

### 3.4. Correlation Analysis between MRS Metabolites and Cognitive Tests with Dosimetric Parameters

**Figure 5:**
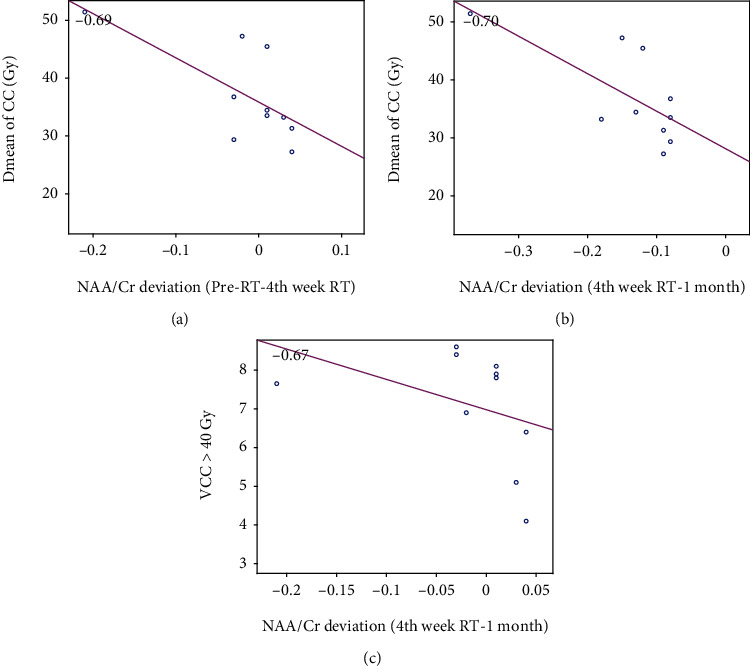
Correlation of the alteration in NAA/Cr between pre-RT and the 4^th^ week of RT with *D*_mean_ of CC (a), NAA/Cr between the 4^th^ week of RT and 1 month post-RT with *D*_mean_ of CC (b), and NAA/Cr between the 4^th^ week of RT and 1-month post-RT with the mean volume of the CC received dose more than 40 Gy (*V*_CC_ > 40 Gy) (c).

The correlation between metabolites, ACE-R, and MoCA average scores with dosimetric parameters of the CC was measured as follows: *D*_mean_ of the CC had a negative correlation with the deviation of NAA/Cr between pre-RT and 4^th^ week of RT (Pearson's correlation coefficient = −0.69, *P* value = 0.024; Figure 5(a)). *D*_mean_ of the CC had a negative correlation with the deviation of NAA/Cr between the 4^th^ week of RT and 1-month post-RT (Pearson's correlation coefficient = −0.70, *P* value = 0.029; Figure 5(b)).

The mean volume of CC received dose higher than 40 Gy (*V*_CC_ > 40Gy) had a negative correlation with the deviation of NAA/Cr between the 4^th^ week of RT and 1-month post-RT (Pearson's correlation coefficient = −0.67, *P* value = 0.035; Figure 5(c)).

It is worth to be noted that the red line in all of the above-mentioned figures represents the best fitted line to the plotted points. Moreover, the represented correlation value is a measure of the level of reliability of this linear relationship, and the relevant *P* value is a measure of uncertainty of this correlation. According to these points, the above reported *P* values prove a high level of reliability to the plotted lines. However, it should be emphasized that more samples are needed to achieve more precise conclusion.

### 3.5. Correlation Analysis between MRS Metabolites with Cognitive Tests

**Figure 6:**
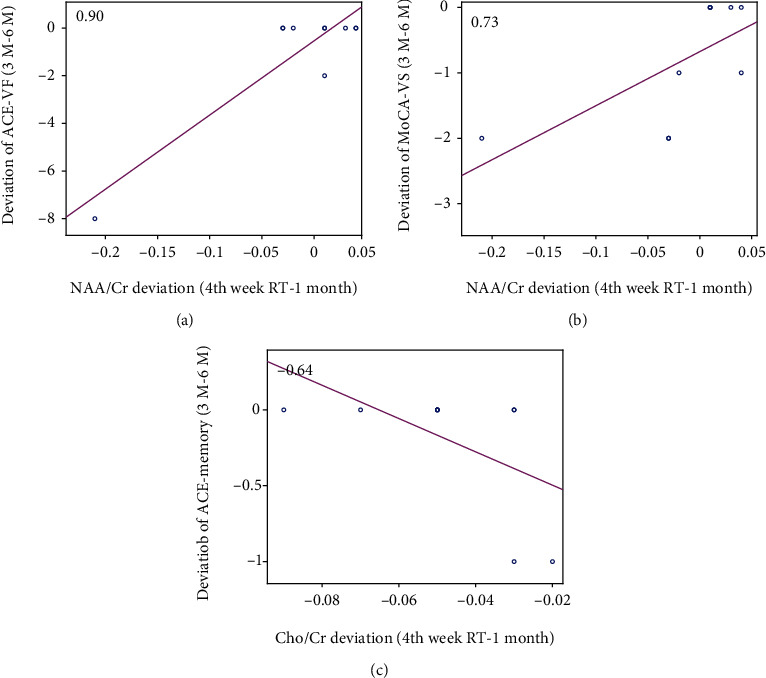
Correlation between alteration in NAA/Cr between the 4^th^ week of RT and 1-month post-RT and deviation of verbal fluency score of ACE-R test (ACE-VF) at the 6-month from the 3-month post-RT (a), between alteration in NAA/Cr between the 4^th^ week of RT and 1 month post-RT and the alteration in visuospatial score of MoCA test (MoCA-VS) between the 3-month and 6-month post-RT (b), and between alteration in NAA/Cr between the 4^th^ week of RT and 1-month post-RT and the alteration in memory section score of ACE-R test (ACE-memory) between the 3-month and 6-month post-RT (c).

The results showed no significant correlation between metabolites ratio including NAA/Cr, Cho/Cr with the overall scores of MoCA, and ACE-R in any time point. However, a significant correlation was observed between the deviation of some metabolite's ratio and the score of cognitive tests subdomains. Namely, the deviation of NAA/Cr at 1-month post-RT from the 4^th^ week of RT showed a positive correlation with the deviation of verbal fluency score of ACE-R test at the 6-month from the 3-month post-RT (Pearson's correlation coefficient = 0.90, *P* value = 0.0003; Figure 6(a)). The alteration in NAA/Cr between the 4^th^ week of RT and 1-month post-RT showed a positive correlation with the alteration in the visuospatial score of the MoCA test between the 3-month and 6^−^month post-RT (Pearson's correlation coefficient = 0.73, *P* value = 0.017; Figure 6(b)). The deviation of Cho/Cr at 1-month post-RT from 4^th^ week of RT showed a negative correlation with the alteration in memory section score of ACE-R test between the 3-month and 6-month post-RT (Pearson's correlation coefficient = 0.64, *P* value = 0.004; Figure 6(c)).

The same as Figure 5, the represented correlations prove a high level of reliability for the observed linear relationships.

## 4. Discussion

We prospectively performed MR spectroscopy and cognitive assessments on patients with low-grade glioma after radiation therapy to assess the radiation-induced injury of the corpus callosum as a critical structure involved in cognitive functions [36]. It was demonstrated that the alteration in MRS metabolites occurred earlier than the cognitive assessments measured by ACE-R (Addenbrooke Cognitive Examination-Revised Version) and MoCA (Montreal Cognitive Assessment), which can establish a useful predictor of cognitive declines in the early delayed phase and may be considered a clinical gold standard of cognitive impairment [38].

Time-trending variations in MRS metabolites in this study confirmed the hypothesis that the microstructural metabolic changes in CC can predict cognitive declines at the early delayed phase. The findings demonstrated that after brain radiation, a significant decline in NAA/Cr continued from the 4^th^ week of RT up to 6-month post-RT was in agreement with other studies reporting radiation-induced injury detectable by MRS metabolites [18, 20, 37, 45–49]. NAA is predominantly overstated in neuronal structures [45]; therefore, a decrease in the NAA concentration has been attributed more likely to neuronal dysfunction rather than neuronal loss [46, 47]. In addition, this study was carried out in regions remote from the tumor, where the decline observed in NAA ensured that the degeneration is not associated with the Wallerian effect [26]. In addition, there are some reports that question the stability of creatine in tumors, hypoxia, and other confounding factors [33, 35]. Therefore, with the adherence to similar studies, the decrease in the NAA/Cr ratio is most likely due to neuronal damage, neuronal cell death due to apoptosis, and neuronal dysfunction secondary to the irradiation rather than an elevation in the creatine itself, which also would result in a decreased NAA/Cr ratio [50]. The decline of NAA could be secondary to other radiation neurotoxicity effects, such as blood-brain-barrier break down or white matter demyelination which may occur with a prescription dose of 54 Gy. It can be confirmed by the fact that blood-brain barrier breakdown occurs early with doses as small as 30 Gy, resulting in abnormal entry of chemical compounds from the blood into the brain tissue [2].

We observed a significant unremitting decline in the Cho/Cr ratio from the fourth week of RT up to 6-month post-RT [18, 20, 37, 45–49]. It has been suggested that the decrease in Cho seen in normal-appearing white matter might be due to membrane injury in the myelin or the myelin-producing oligodendrocytes, accompanied by impaired tissue perfusion [31, 46]. A significant increase in the NAA/Cho ratio was also observed in consecutive time points only at the early phase. Other studies observed a significant increase in NAA/Cho in a normal brain tissue at an early delayed phase. Thus, elevated NAA/Cho ratios may be due to early radiation-induced inflammation, demyelination, or gliosis, which can decrease over time [18, 20].

Alterations in metabolites as an established evidence of microstructural damage in the normal appearing white matter have been reported to be significantly associated with cognitive worsening after brain RT in other studies [9, 51]. Therefore, metabolic variations of the CC in this study can be an evidence of cognitive dysfunctions. In our study, significant changes in patient's MoCA and ACE-R scores at 3 and 6-month post-RT were observed, while an abnormal score was not reported for all patients. For both MoCA and ACE-R, the lower abnormal score was only seen for patients with lower baseline scores, similar to Brown et al.'s study [52].

Our study demonstrated a decline in language and verbal fluency and visuospatial and memory scores specifically at 3- and 6-month post-RT. Although none of these declines stayed significant after Bonferroni adjustment, they still show a trend of declines. It is has been shown that the variation in the microstructure of the CC can strongly affect visuospatial perception and verbal fluency tasks, as well as other cognitive functions such as memory which may result in serious cognitive disorders [53–55]. The positive correlation of the posterior callosum with verbal fluency and language lateralization and also the anterior callosum with visuospatial ability has been demonstrated [56–58].

The correlation between the variations in MoCA and ACE-R subdomains versus the variations in metabolites was investigated as well. The alteration in NAA/Cr between the 4th week of RT and 1-month post-RT showed a positive correlation with the alteration of the verbal fluency score of the ACE-R test between the 3-month and 6-month post-RT. Several studies showed that both NAA/Cr and Cho/Cr ratios are related to cognitive variables and correlated with verbal fluency in neurocognitive diseases [59–61]. The deviation of NAA/Cr at 1-month post-RT from 4th week of RT showed a positive correlation with the alteration in the visuospatial score of ACE-R test between the 3-month and the 6-month post-RT. The splenium of CC interconnects regions of the parietal and temporal cortices [62]; therefore, it is expected that posterior CC is involved in mediating the visuospatial and language functions. [63, 64]. Also, the alteration of Cho/Cr between the 4^th^ week of RT and 1-month post-RT showed a negative correlation with the alteration of the memory section score of the ACE-R test between the 3-month and the 6-month post-RT. This is in agreement with the fact that the disruption in callosal function can negatively impact memory in individuals with a variety of neurological disorders. For example, structural callosal damage in patients with multiple sclerosis is associated with impaired list learning [65]. The corpus callosum plays an important, yet indirect, role in facilitation of the memory [66]. It is consented that higher NAA/Cr as well as Cho/Cr ratios are associated with a better performance on logical as well as verbal memory. Cho/Cr is also correlated with the visual, the auditory, and verbal memory tests [60].

The imperative role of NAA in neural tissue has been established at more macroscopic levels, for example, toward understanding of the potential biochemical foundations of cognitive ability [67]. Studies have suggested that higher NAA and choline levels are associated with better cognitive function [60, 68]. However, the higher creatine levels might better explain the correlations between NAA/Cr and Cho/Cr ratios and cognitive variables compared to absolute NAA or choline levels [69]. Conventionally, creatine levels are commonly used as the denominator and are also considered to change slightly as a result of specific diseases [69]. As a substantial marker of neuronal integrity, the NAA/Cr ratio has been assessed instead of absolute NAA. Nonetheless, increases in creatine would have the same observation, and this issue is still quite controversial [22].

Both ACE-R and MoCA are reported to have an acceptable sensitivity and specificity in diagnosing cognitive dysfunctions [70]. Comparing MoCA, some studies achieved higher sensitivity but lower specificity for MoCA in screening cognitive impairments and have been regarded more suitable than ACE-R. In contrast, some studies suggested that ACE-R held sensitivity and specificity values higher than MoCA in all the subdomains [71]. This study presented that ACE-R may have more potential to provide evidence of cognitive decline in both language and verbal fluency functions. The reason may be due to the fact that the ACE-R tool has several extra subdomains than MoCA which may be exhausting for the patients to accomplish. In memory subdomain, overall, ACE-R presented a decline earlier than MoCA for the majority of the patients. The decline in the visuospatial function was presented by MoCA for more patients than ACE-R. It may be due to the fact that this subdomain is more complicated in the MoCA test and many patients are not able or do not bear to accomplish it properly.

One of the parameters that may affect the white matter and consequently cognitive impairments is the delivered dose [8, 32, 50, 72] to the critical structures. In our study, the mean dose delivered to the CC showed a negative correlation with the deviation of NAA/Cr between pre-RT and 4^th^ week of RT and a negative correlation with the deviation of NAA/Cr between the 4^th^ week of RT and 1-month post-RT. The dose dependence of the metabolic deviance in normal appearing brain tissue is a controversial issue confirmed by some studies, yet rejected by others [18, 52].

It should be emphasized that the tumor progression, extended to the CC periphery, was seen in one of the patients with an abnormal cognitive test score in both 3- and 6-months follow-ups. Tumor progression after RT has been reported to adversely affect cognition, confounding imaging and the measurement of radiation-induced cognitive declines [52]. Other confounding factors that may influence cognitive impairments such as fractionation regimen, histology, KPS score, and antiepileptic medications [10] were assumed constant across the population under the study. A major limitation of this study was the relative small number of patients, which is usually the case in prospective longitudinal studies, especially when imaging protocols with long scan time such as quantitative MRI are used [19, 72].

## 5. Conclusion

This study specified a significant variation of MRS metabolite values in the corpus callosum starting at early phases after RT of LGG patients, which remained evident for early delayed and delayed phases. Since the corpus callosum plays a critical role in most of the cognitive pathways, such early delayed variation of metabolites can be considered a more sensitive predictor of long-term detrimental effects of radiotherapy on cognitive functions, in comparison with ACE-R or MoCA cognitive scores. The correlation between subdomain scores and metabolite variation can be considered important in the conduction of a plan to evaluate the cognitive functions associated to the corpus callosum damage after radiotherapy. The results of this study complement the earlier reports demonstrating the effect of radiotherapy and dose dependency on white matter morphology and integrity, emphasizing the importance of dose sparing protocol for the corpus callosum during the treatment planning. In addition, this study suggests that the MRS study of early variations in NAA/Cr and Cho/Cr in the corpus callosum may be exploited to modify the treatment plans for the patients and to design more sensitive cognitive assessment tools. The results of this research introduce reliable quantitative imaging biomarkers that can predict a cognitive decline in LGG patients before it manifests at the late delayed stages.

## Figures and Tables

**Table 1:** A summary of the characteristics of the patients analyzed.

Number	Gender/age	KPS^a^	Histology	Tumor size (mm)	Antiepileptic drug	Location
1	M^b^/19	100	Oligodendroglioma-II	53 × 45	Sodium valproate	LT^d^
2	F^c^/22	100	Oligodendroglioma-II	42 × 31	Sodium valproate	LF^e^
3	M/35	100	Oligodendroglioma-II	37 × 30	Sodium valproate	LT
4	M/53	95	Oligodendroglioma-II	58 × 46	Sodium valproate	LT
5	F/41	100	Oligodendroglioma-II	38 × 32	Sodium valproate	LF
6	F/42	100	Oligodendroglioma-II	35 × 33	Sodium valproate	LF
7	F/25	100	Oligodendroglioma-II	45 × 23	Sodium valproate	LT
8	F/26	90	Oligodendroglioma-II	55 × 46	Sodium valproate	RP^f^
9	M/36	100	Oligodendroglioma-II	64 × 65	Sodium valproate	LF
10	F/54	100	Oligodendroglioma-II	48 × 43	Sodium valproate	LF

a: Karnofsky performance status; b: Male; c: Female; d: Left temporal; e: Left frontal; f: Right parietal. It has been added.

**Table 2:** The number of cases for which a decline was detected by the subdomains of cognitive tests (ACE-R and MoCA) at different time points.

	Number of impaired patients				
Cognitive domain	Cognitive test	4^th^ week	1 M post-RT	3-month post-RT	6-month post-RT
Language and verbal fluency	ACE-R	2	2	1	3
	MoCA	0	0	0	3
	Total	2	2	1	4

Memory	ACE-R	0	0	2	4
	MoCA	1	0	0	3
	Total	1	0	2	4

Visuospatial	ACE-R	0	0	0	3
	MoCA	0	0	0	4
	Total	0	0	0	4

## Data Availability

The MRS data used to support the findings of this study are available from the corresponding author upon request.
